# Supplementary material for: Pumilio-dependent localization of mRNAs at the cell front coordinates multiple pathways required for chemotaxis
Source: Nat Commun. 2017 Nov 8;8:1366. doi: 10.1038/s41467-017-01536-x (PMC5678099; doi:10.1038/s41467-017-01536-x)
Supplement: Supplementary file 1 — Supplementary Information [file 41467_2017_1536_MOESM1_ESM.pdf]

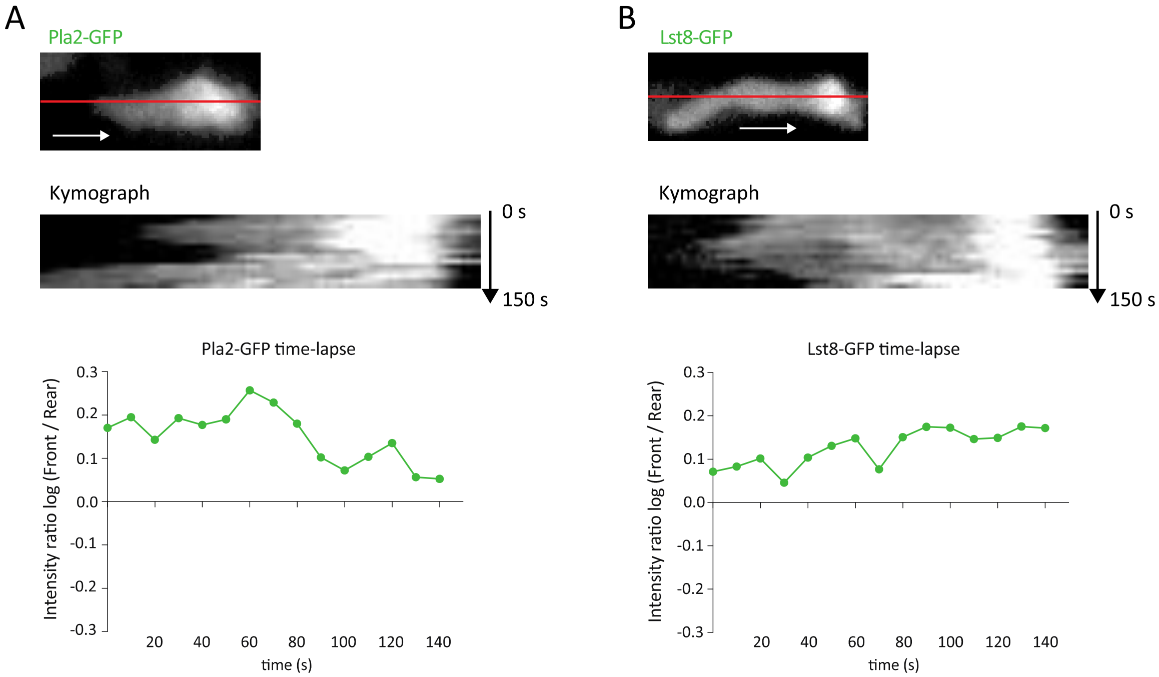

Supplementary Figure 1: Pla2 and Lst8 are enriched in the dynamic pseudopod. Kymographs measured along the depicted red line showing Pla2- and Lst8-GFP enrichment in the dynamic pseudopod of a migrating cell (A and B respectively). White arrow indicates direction of migration. Quantification of the fluorescence intensity ratio between the front and rear of the migrating cell measured as in Fig. 1B (logF/R). The grey bar indicates values equivalent to symmetric localization (between log(0.9) and log(1.1)).

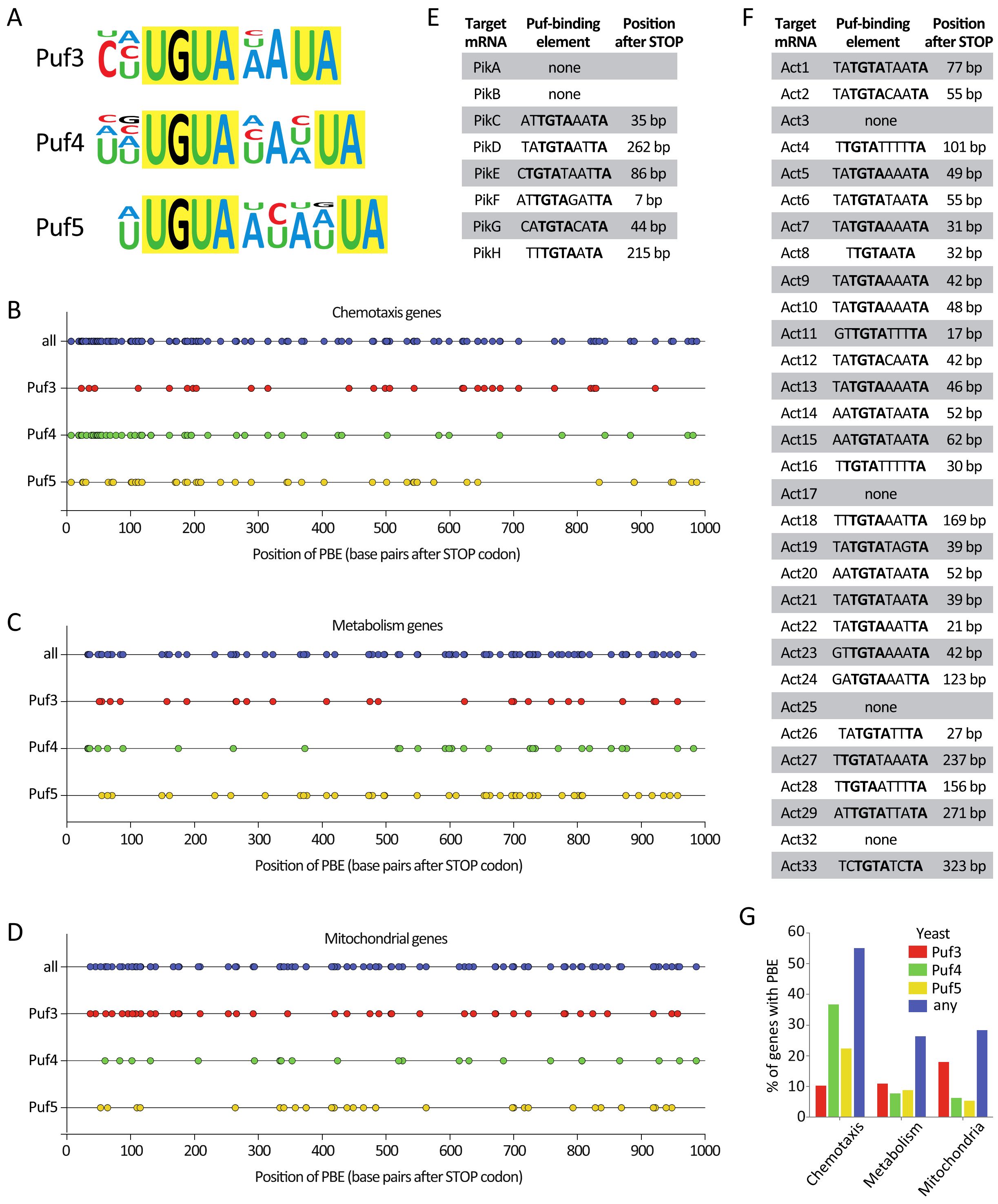

E

Target mRNA	Puf-binding element	Position after STOP
PikA	none	
PikB	none	
PikC	ATTGTAAATA	35 bp
PikD	TATGTAATTA	262 bp
PikE	CTGTATAATTA	86 bp
PikF	ATTGTAGATTA	7 bp
PikG	CATGTACATA	44 bp
PikH	TTTGTAATA	215 bp

F

Target mRNA	Puf-binding element	Position after STOP
Act1	TATGTATAATA	77 bp
Act2	TATGTACAATA	55 bp
Act3	none	
Act4	TTGTATTTTTA	101 bp
Act5	TATGTAAAATA	49 bp
Act6	TATGTATAATA	55 bp
Act7	TATGTAAAATA	31 bp
Act8	TTGTAATA	32 bp
Act9	TATGTAAAATA	42 bp
Act10	TATGTAAAATA	48 bp
Act11	GTTGTATTTTA	17 bp
Act12	TATGTACAATA	42 bp
Act13	TATGTAAAATA	46 bp
Act14	AATGTATAATA	52 bp
Act15	AATGTATAATA	62 bp
Act16	TTGTATTTTTA	30 bp
Act17	none	
Act18	TTTGTAAATTA	169 bp
Act19	TATGTATAGTA	39 bp
Act20	AATGTATAATA	52 bp
Act21	TATGTATAATA	39 bp
Act22	TATGTAAATTA	21 bp
Act23	GTTGTAAAATA	42 bp
Act24	GATGTAAATTA	123 bp
Act25	none	
Act26	TATGTATTTA	27 bp
Act27	TTGTATAAATA	237 bp
Act28	TTGTAATTTTA	156 bp
Act29	ATTGTATTATA	271 bp
Act32	none	
Act33	TCTGTATCTA	323 bp

Supplementary Figure 2: Pumilio-binding elements (PBE) are abundant among chemotaxis genes in *Dictyostelium discoideum*. A. The PBE consensus recognition-sites for yeast Puf3, Puf4 and Puf5 [3]. A, Adenine; C, Cytosine; G, Guanine; T, Thymine.

Letter height represents the likelihood of a base at a given position. B - D: Position of PBEs among genes annotated as function in “Chemotaxis” (D, n = 97), “Metabolism” (E, n = 57) and “Mitochondria” (F, n = 93). E. PBEs in the 3’-UTR of PI3-Kinase genes. F. PBEs in the 3’-UTR of actin genes. G. Percentage of genes with PBEs within 330 base pairs after the STOP codon in these 3 functional categories.

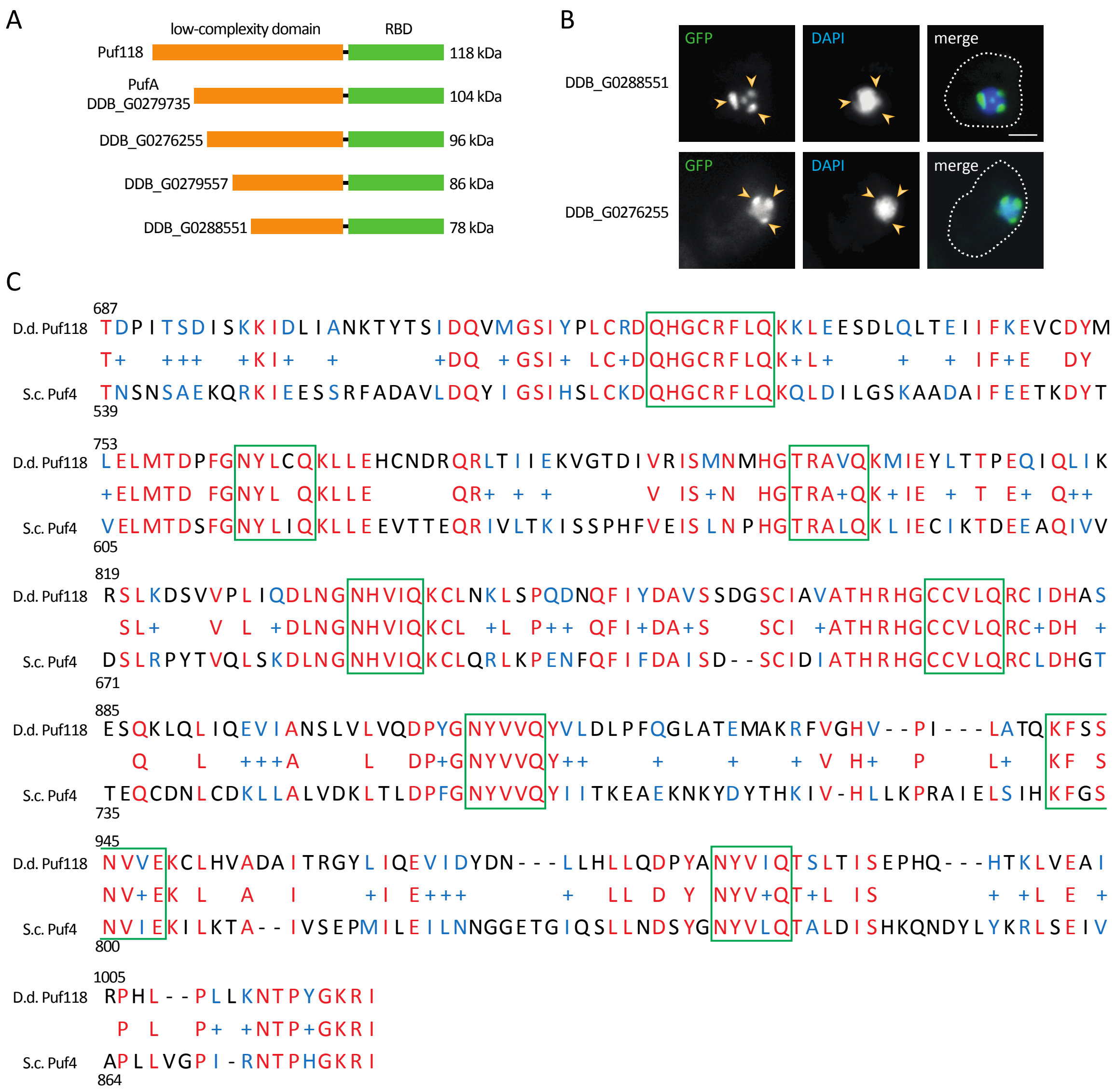

Supplementary Figure 3: The five Pumilio/Puf homologues in *Dictyostelium discoideum*. A. Domain structure of the five Puf homologues. B. Localization of GFP-tagged DDB_G0288551 and DDB_G0276255, and nuclear DAPI stain in vegetative cells. Arrowheads point at GFP-rich patches representing the nucleolus, as shown by exclusion of the DAPI stain. Scale bar, 5 μm. C. Protein sequence alignment of *D. discoideum* Puf118 and *S. cerevisiae* Puf4. The green boxes highlight the residues necessary for mRNA target recognition. Identical residues are shown in red, similar residues are shown in blue.

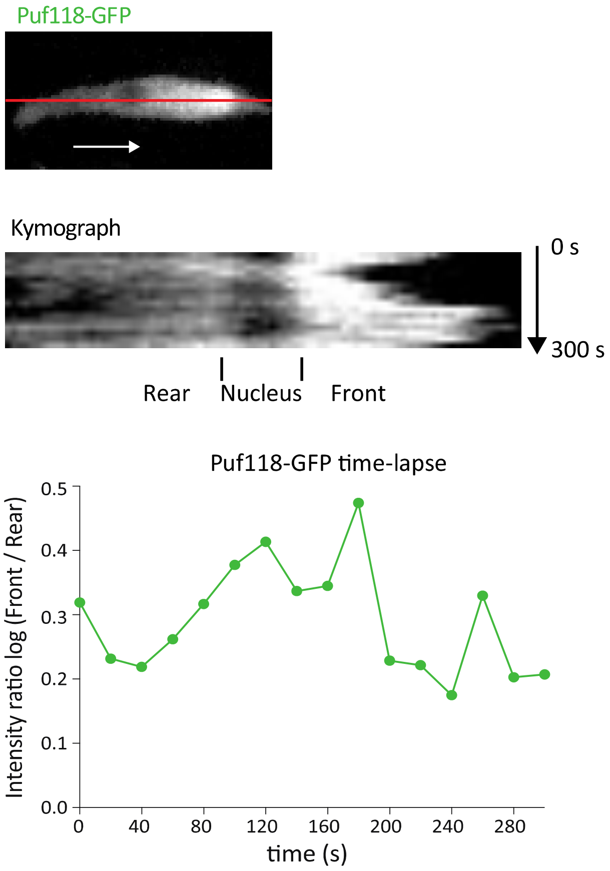

Supplementary Figure 4: Puf118 is enriched in the dynamic pseudopod. A. Kymograph measured along the depicted red line showing Puf118-GFP enrichment in the dynamic pseudopod of a migrating cell (see Fig. 2B). B. Quantification of the Puf118-GFP fluorescence intensity ratio between the front and rear of the migrating cell in (A). (logF/R). The grey bar indicates values equivalent to symmetric localization (between log(0.9) and log(1.1).

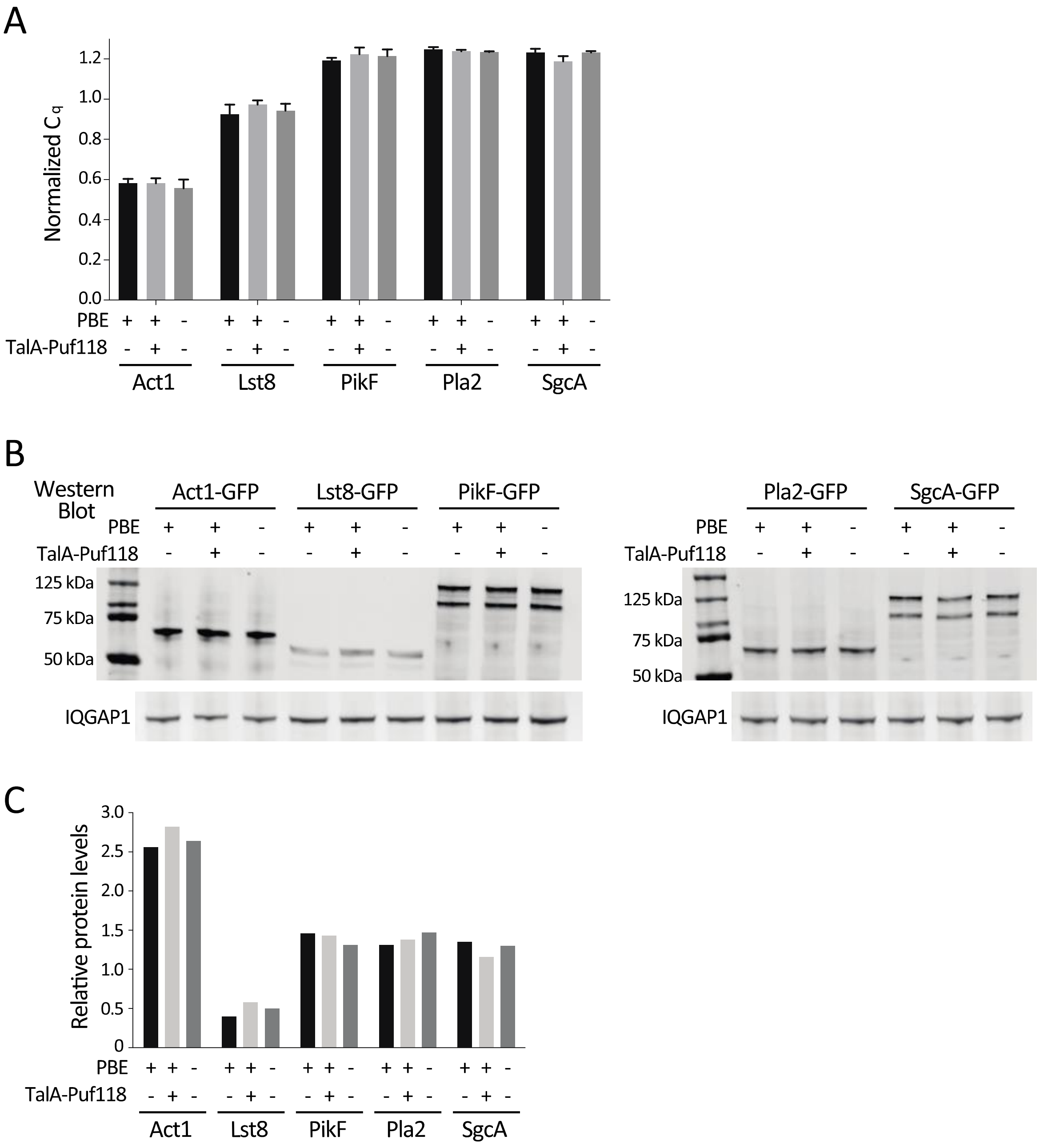

Supplementary Figure 5: Chemotaxis pathway mRNA and protein abundance are not affected by the -PBE mutations and by TalA-Puf118 co-expression. A. C_q-values of RT-qPCR detecting total mRNA for the indicated genes in wild-type cells expressing +PBE or -PBE variants, with or without co-expressed TalA-Puf118. No-RT negative controls were included and were undetectable. All C_q-values were normalized to RT-qPCR levels of the control gene ScdA included in each sample. B. Protein levels

of indicated genes in wild-type cells expressing +PBE or -PBE variants, with or without co-expressed TalA-Puf118. IQGAP1 was used as a loading control. C. Quantification of (B). Values were normalized to the IQGAP1 intensity for each lane.

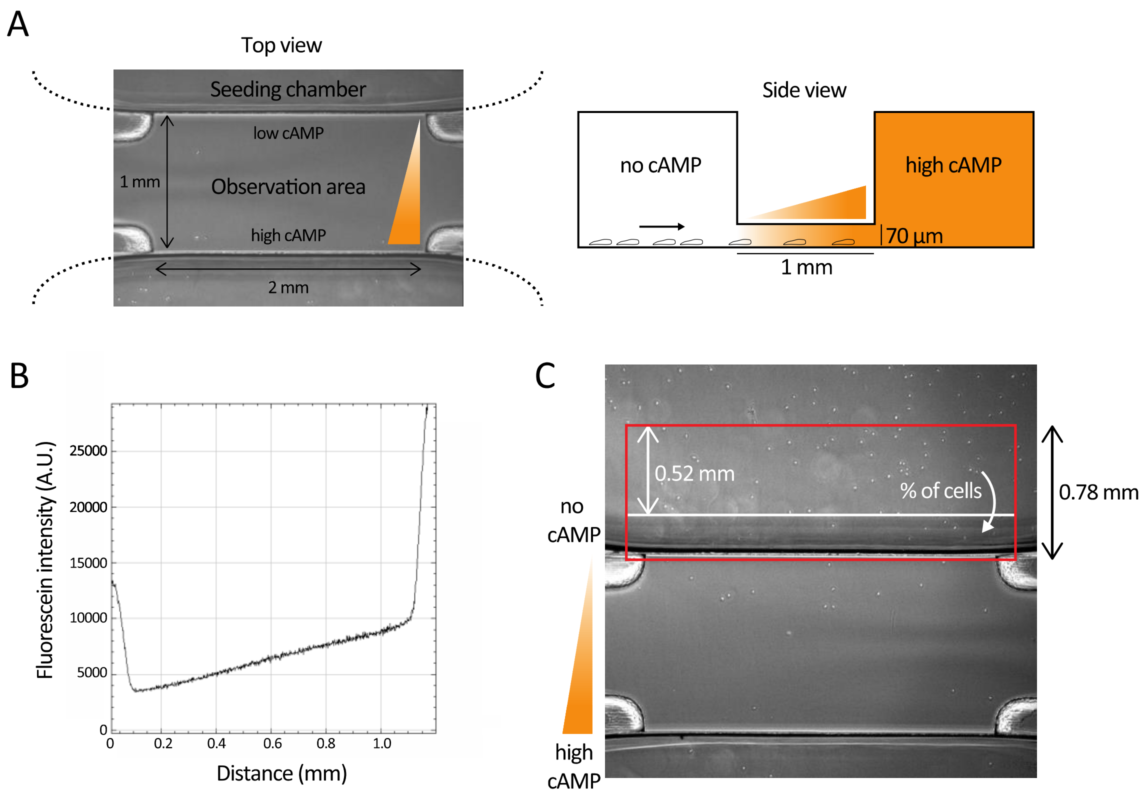

Supplementary Figure 6: The Ibidi µ-Slide chemotaxis chamber. A. Design of the Ibidi µ-Slide chemotaxis chambers. Top view showing the observation chamber where the cAMP gradient is formed and adjacent reservoirs (seeding chamber for cell loading and cAMP reservoir). Side view shows the principle of gradient formation within the chamber. B. Gradient formation was tested by addition of Fluorescein instead of cAMP. Fluorescence was measured at 30 min after Fluorescein addition. C. Depiction of how chemotaxis outside the observation chamber was quantified (Figs. 3D, 4C). We determined the percentage of cells moving from the top 2/3 of the red rectangle (2.29 x 0.78 mm) into the lower 1/3 by crossing the white line over the course of a 180 min movie.

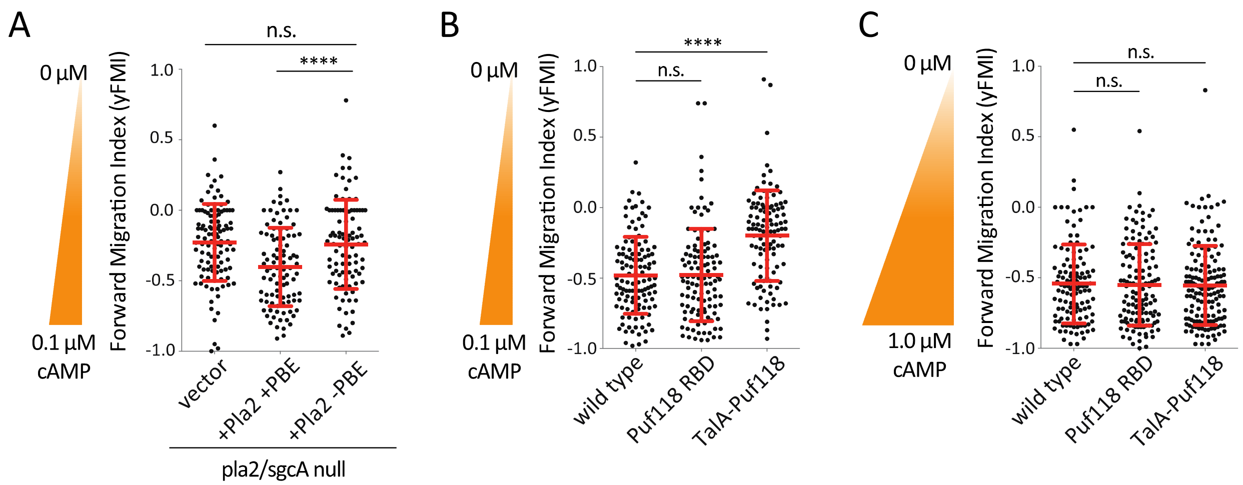

Supplementary Fig. 7: Quantification of chemotaxis efficiency. A-C. Quantifications of the Forward Migration Index (yFMI) of the chemotaxis tracks in Fig. 3F, 4E and 4G. Mann-Whitney test on $n \geq 95$ cells from ≥ 3 independent experiments: **** $p < 0.0001$, n.s. not significant.

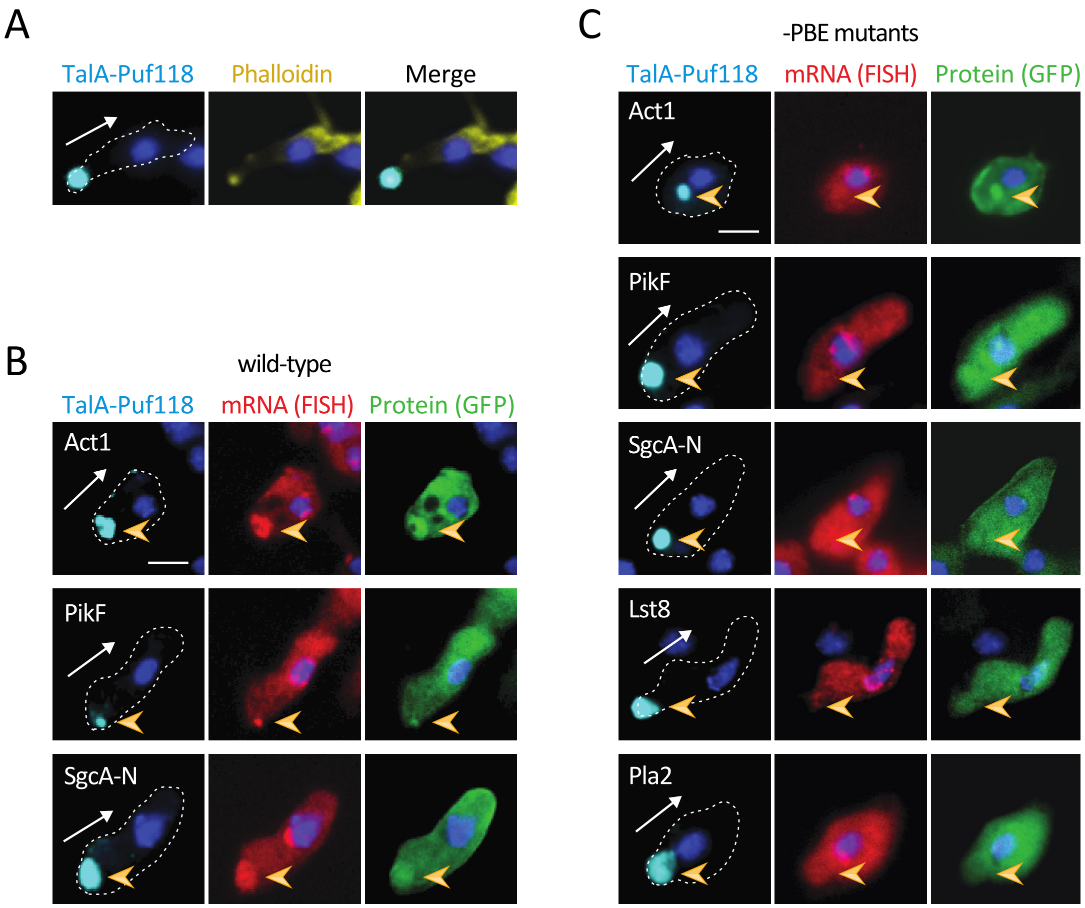

Supplementary Fig. 8: Chemotaxis pathway mRNAs co-localize with myc-TalA-Puf118 in a PBE-dependent manner. A. Localization of myc-tagged TalA-Puf118 to the cell rear. Arrow indicates orientation of cell polarity defined by F-actin stain (Alexa Fluor 647-conjugated Phalloidin). DNA (nucleus) was stained with DAPI. B-C. Co-localization of TalA-Puf118 with GFP-Act1, -Lst8, -PikF, -Pla2 and -SgcA-N and the corresponding mRNAs expressed as +PBE (B) or -PBE (C) form in cells of natural chemotactic streams (arrowheads). Arrow indicates orientation of cell polarity defined by TalA-Puf118 localization. Differences in morphology are due to the fact that expression of the TalA-Puf118 construct perturbs overall cell polarity. Note that the Act1 -PBE protein, unlike the mRNA, co-localizes with the TalA-Puf118 construct despite the disrupted PBE. This is explained by the fact that Talin A itself is an actin-binding protein. Scale bar, 5 μm.

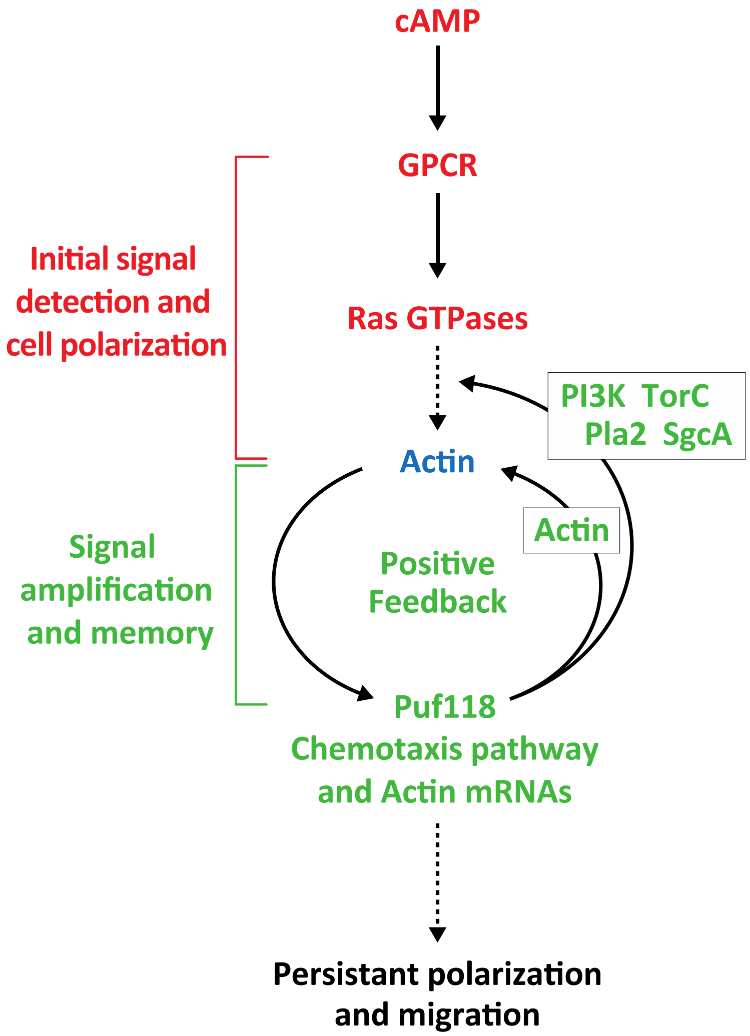

Supplementary Fig. 9: Model of signaling pathways controlling chemotaxis in *Dictyostelium discoideum*. Cyclic AMP is sensed at the cell front by GPCRs and the signal is propagated to Ras GTPases, causing the rapid, polarized assembly of actin at the cell front. Puf118 and its bound chemotaxis pathway and Act1 mRNAs is recruited to the cell front in an F-actin-dependent manner. mRNA translation at the cell front provides a positive feedback-loop to: 1) maintain localized actin assembly, and 2) locally amplify the weak initial polarization and memory of the direction of migration, thus leading to persistent chemotaxis.

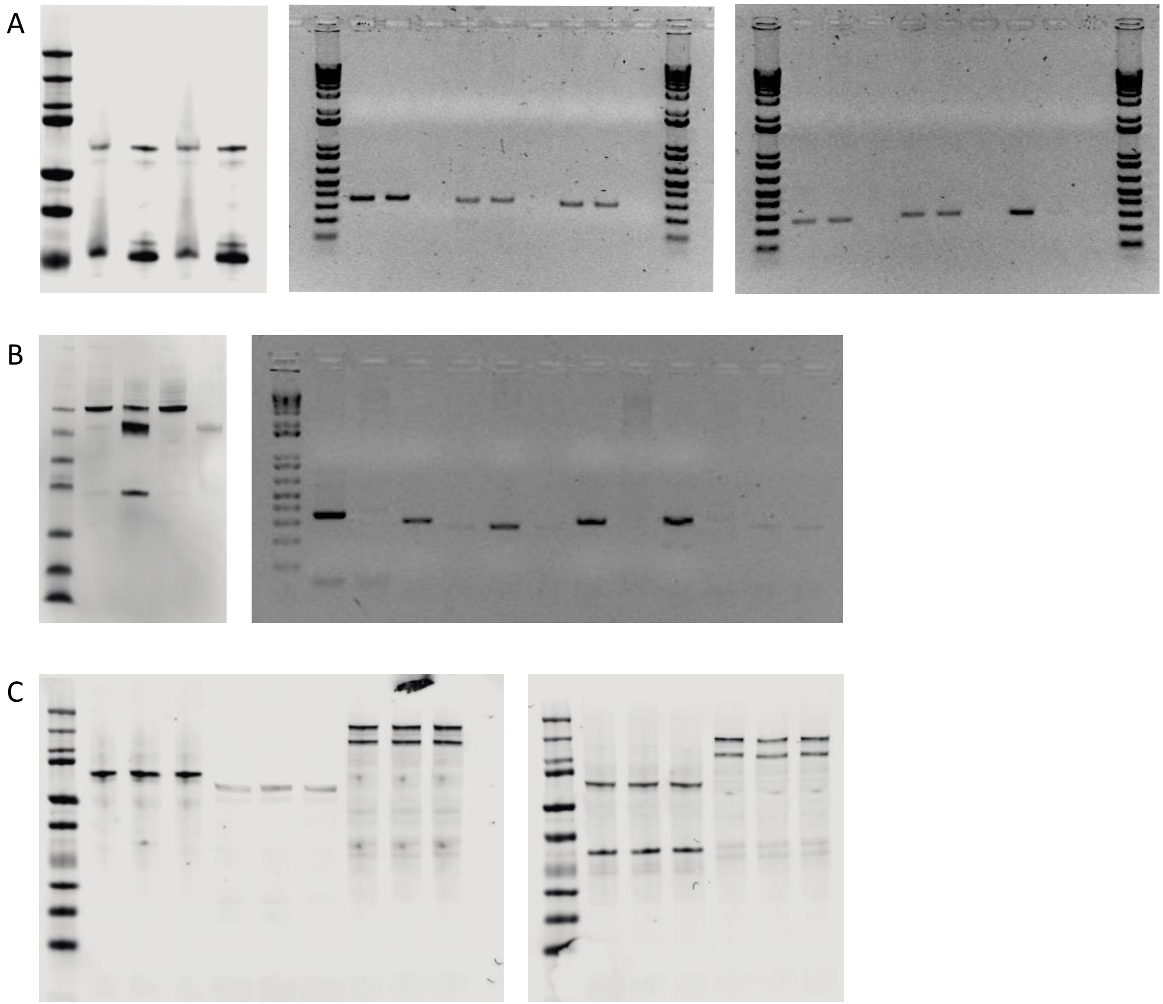

Supplementary Fig. 10: Uncropped blots from all Figures of this study. A. Western Blot (left) and DNA-Agarose gels (middle and right) as in Fig. 2D. B. DNA-Agarose gel as in Fig. 4A. C. Western Blots as in Supplementary Fig. 5.

Chemotaxis gene	Sequence	Position		Chemotaxis gene	Sequence	Position
DDB_G0270774	TTGTATCAATA	172		lst8	ATGTATTATTA	23
DDB_G0275303	ATGTATTAATA	112		mhkA	TTTGTATTTTA	279
DDB_G0278215	ATGTAAAAGTA	186		mpl1	ATGTATTGATA	264
DDB_G0292988	ATTGTAAAATA	68		myoC	ATTGTAATTTA	132
DDB_G0293176	TTGTAATAATA	73		pakA	ATGTATTAATA	170
abcB1	TTTGTAAATTA	161		pakC	TTGTAATAATA	108
abcE1	TTTGTAATTA	198		phg2	ATGTATTTTTA	118
actin*				piaA	TTGTATTAATA	71
carA-1	TCTGTACAATA	118		Pi3Ks*		
carA-2	TCTGTACAATA	118		pla2	ATTGTATTTTA	24
dagA	CTTGTAAATA	23		rapA	TATGTATATA	289
erkB	AATGTAAAATA	189		rasD	TTTGTATAATA	132
gcA	GTGTAATATTA	7		secG	TTTGTATTTA	203
gpaD	TTGTAATAATA	108		sgcA	ATTGTAAAATA	86
gpbA	CTTGTATAATA	195		shkA	AATGTATGATA	315
gpgA	ATTGTACATTA	19		tor	TTTGTAAAATA	185
grlE	TTGTAAAATTA	26		yakA	ATTGTAAATTA	266
limE	TTTGTACACTA	19		yelA	TTTGTAAAATA	107
limD1	AATGTATATA	112				

Supplementary Table 1: Full list of PBE-containing chemotaxis genes. All genes annotated with "chemotaxis" that contain a PBE within 330 base pairs after the STOP codon. For genes with multiple PBEs, the first one is shown. Asterisks indicate that

multiple genes exists and the detailed analysis of their PBEs is shown in Supplementary Fig. 2 E-F.

Oligo Name	Sequence	Target ORF	Position on target ORF	Method used for	Product size
Act1 f	ACTTCGTGTTGCTCCAGAGG	Act1	282 bp	RT-qPCR	138 bp
Act1 r	ACAGCTTGAATGGCAACATAC	Act1	399 bp	RT-qPCR	
Lst8 f	CCACCCAGTGGTGGATGTTA	Lst8	58 bp	RT-qPCR	110 bp
Lst8 r	GTTTGTGGGTTACCTGCTGC	Lst8	310 bp	RT-qPCR	
PikF f	CACCATCAACTGAAACCTGTGG	PikF	3306 bp	RT-qPCR	112 bp
Pikf r	ATTGGTTCACCAACGGGGTC	PikF	3398 bp	RT-qPCR	
Pla2 f	GGTTCCCTTTGGGACGAAGT	Pla2	347 bp	RT-qPCR	117 bp
Pla2 r	GGTATCAGCTCCATCTGGTGA	Pla2	443 bp	RT-qPCR	
ScdA f	CGTATTCCAGGTGATGCCGA	ScdA	1494 bp	RT-qPCR	100 bp
ScdA r	ACCAACCCAAACTGAATGCC	ScdA	1574 bp	RT-qPCR	
SgcA f	TCCAAAAATTCACTTTCTCACAACC	SgcA	2142 bp	RT-qPCR	137 bp
SgcA r	TGGATGATGTGGTGGTTTGGAT	SgcA	3433 bp	RT-qPCR	
RT Act1 f	GAAGCTGAAATGCAAACTGCAGC	Act1	673 bp	RNA IP	346 bp
RT Act1 r	CAGAGTATTTACGTTCTGGTGGTGC	Act1	994 bp	RNA IP	
RT Lst8 f	GGAGATTGGGTGAAGATGATAC	Lst8	814 bp	RNA IP	342 bp
RT Lst8 r	GTAAATGCTGTTGCTTTAAATG	Lst8	1134 bp	RNA IP	
RT PikF f	AATGTTGACCCCGTTGGTGA	PikF	3392 bp	RNA IP	304 bp
RT PikF r	AGTCGGGGTCAGATTGATTTCT	PikF	3674 bp	RNA IP	
RT Pla2 f	CACCAGATGGAGCTGATACCC	Pla2	444 bp	RNA IP	272 bp
RT Pla2 r	AGCTGCTGGAATACTTGACG	Pla2	696 bp	RNA IP	
RT ScdA f	ACCGACCAAAGATCTCCACG	ScdA	1737 bp	RNA IP	308 bp
RT ScdA r	TGAGCACGAGTGAATGATGGT	ScdA	2024 bp	RNA IP	
RT SgcA f	TGCTGTCACTGTCCCCAAAT	SgcA	9260 bp	RNA IP	301 bp
RT SgcA r	GGTAGTGGTTCACCAAGTGGT	SgcA	9540 bp	RNA IP	

Supplementary Table 2: List of Oligos used in this study.

Supplementary Note 1

Pumilio/Puf-like proteins in *Dictyostelium discoideum*

Other than Puf118 (DDB_G0289987; 118 kDa), four genes have been annotated as Pumilio-related on Dictybase (www.dictybase.org; Supplementary Fig. 2): PufA (annotated as 104 kDa), DDB_G0276255 (96 kDa), DDB_G0288551 (78 kDa) and DDB_G0279557 (86 kDa; “Puf86”).

PufA is the closest homologue of yeast Puf3, which specifically binds mitochondrial mRNAs and targets them to the mitochondria [1]. PBEs predicted to be recognized by Puf3 were enriched among mitochondrial genes of *Dictyostelium discoideum* (Supplementary Fig. 2F,G). Note that the protein is likely larger than 104 kDa (as annotated in Dictybase), due to an incomplete sequence contingent that left a gap of unknown size in the DNA sequence (http://dictybase.org/gene/DDB_G0279735).

DDB_G0288551 and DDB_G0276255 were predicted to localize to the nucleolus (http://www.compbio.dundee.ac.uk/www-nod/). This was confirmed by expression of GFP-tagged proteins, which showed localization of both proteins to regions within the nucleus where DAPI-stained DNA was excluded (Supplementary Fig. 3B), which is a characteristic of nucleolar proteins [2].

DDB_G0279557 is predicted to be expressed late in development ([3], www.dictyExpress.org) and, accordingly, we did not detect any GFP-tagged protein in the vegetative stage and in early development (MH, unpublished result).

Thus, Puf118, which we named after its apparent molecular mass of 118 kDa, was the best candidate for regulating chemotactic processes during very early stages of development when mRNA levels of Puf118 peaked and chemotactic streaming starts (6 hrs into development [3], www.dictyExpress.org).

Pumilio-binding elements (PBE) in Actin and PI3-Kinases in *Dictyostelium discoideum*

Dictyostelium discoideum has 31 genes coding for actin, of which 29 genes translate to proteins with amino acid identity higher than 83% (http://dictybase.org/gene/DDB_G0289553). Of these genes, seventeen encode identical proteins (*act1*, *act2*, *act4*, *act5*, *act6*, *act7*, *act8*, *act9*, *act11*, *act12*, *act13*, *act14*, *act15*, *act16*, *act19*, *act20*, and *act21*), 3 have minor amino acid differences (*act10*, *act22*, and *act3*), and 6 are more divergent (*act17*, *act18*, *act23*, *act24*, *act32* and *act33*). 27 of the 31 actin genes have a PBE in a very prominent position close to the STOP codon (Supplementary Fig. 2C). Since many actin genes may have arisen by duplication of entire loci [4], the PBEs could have been copied along with the entire 3'-UTR. However, the fact that the exact position of the PBEs within the 3'-UTR and the surrounding sequences differ between the individual actin genes underlines the conservation and functional importance of these PBEs among actin genes. In our study, we focused on Act1 as a representative actin protein.

Dictyostelium discoideum has 8 homologues of mammalian PI3-Kinase, PikA - PikH, most of which act redundantly in chemotaxis [5]. Six of these genes (PikC - PikH) have PBEs close to their STOP codon (Supplementary Fig. 2B). In our study, we focused on PikF as a representative PI3-Kinase.

Supplementary References

1 Garcia-Rodriguez, L. J., Gay, A. C. & Pon, L. A. Puf3p, a Pumilio family RNA binding protein, localizes to mitochondria and regulates mitochondrial biogenesis and motility in budding yeast. *Journal of Cell Biology* **176**, 197-207, doi:10.1083/jcb.200606054 (2007).

2 Balbo, A. & Bozzaro, S. Cloning of Dictyostelium eIF6 (p27(BBP)) and mapping its nucle(ol)ar localization subdomains. *Eur J Cell Biol* **85**, 1069-1078, doi:10.1016/j.ejcb.2006.05.010 (2006).

3 Rosengarten, R. D. *et al.* Leaps and lulls in the developmental transcriptome of Dictyostelium discoideum. *Bmc Genomics* **16**, doi:Artn 294
10.1186/S12864-015-1491-7 (2015).

4 McKeown, M., Hirth, K. P., Edwards, C. & Firtel, R. A. Examination of the regulation of the actin multigene family in Dictyostelium discoideum. *Prog Clin Biol Res* **85 Pt A**, 51-78 (1982).

5 Hoeller, O. & Kay, R. R. Chemotaxis in the absence of PIP3 gradients. *Current Biology* **17**, 813-817, doi:10.1016/j.cub.2007.04.004 (2007).
